# A Challenging Case of Metastatic Intra-Abdominal Synovial Sarcoma with Unusual Immunophenotype and Its Differential Diagnosis

**DOI:** 10.1155/2012/786083

**Published:** 2012-08-27

**Authors:** Yi-Che Changchien, Uhrin Katalin, János Fillinger, László Fónyad, Gergő Papp, Ferenc Salamon, Zoltán Sápi

**Affiliations:** ^1^1st Department of Pathology and Experimental Cancer Research, Semmelweis University, Budapest 1085, Hungary; ^2^Department of Pathology, Kátai Gábor Hospital, Karcag 5300, Hungary; ^3^Department of Pathology, Korányi Hospital, Budapest 1121, Hungary; ^4^Department of Pathology, Uzsoki Hospital, Budapest 1145, Hungary

## Abstract

The primary and metastatic gastrointestinal synovial sarcoma is rare with a wide differential diagnosis. It usually expresses cytokeratins EMA, BCL2 with an occasional CD99, and S100 positivity but not desmin. We present a case of metastatic synovial sarcoma with unusual immunophenotype causing diagnostic challenges. The tumor cells showed focal cytokeratin, EMA, and, unexpectedly, desmin positivity. Additional intranuclear TLE-1 positivity and negativity for CD34 and DOG-1 were also identified. A diagnosis of monophasic synovial sarcoma was confirmed by using FISH break-apart probe. RT-PCR revealed the SYT-SSX1 fusion gene. Intra-abdominal synovial sarcoma, either primary or metastatic, with unusual desmin positivity raises the diagnostic challenge, since a wide range of differential diagnoses could show a similar immunophenotype (leiomyosarcoma, desmoid tumor, myofibroblastic tumor, and rarely GIST etc.). Typical morphology and focal cytokeratin/EMA positivity should alert to this tumor, and FISH and RT-PCR remain the gold standard for the confirmation.

## 1. Introduction

Synovial sarcomas are rare, unique spindle cell tumors probably of mesenchymal cell origin [1]. They occur most commonly in the young patients, representing about 10% of all soft tissue sarcomas and about 15–20% of cases in adolescents and young adults [2]. The peak incidence is before the 5th decade with a slightly male predominance. More than 80% arise in deep soft tissue around the large joint or tendon [3]. The primary gastrointestinal synovial sarcoma is in rare regions [4, 5]. The differential diagnosis is wide since gastrointestinal stromal tumor (GIST), solitary fibrous tumor, myogenic, and neurogenic tumors could have similar, if not identical morphology. It usually expresses cytokeratin, EMA, BCL2 with occasional CD99, and S100 positivity. Among the myogenic markers, calponin and occasional focal positivity of smooth muscle actin can be found but not desmin [6]. We present a case of metastatic synovial sarcoma from a 26-year-old Hungarian male with unusual immunophenotype showing desmin positivity, which raises diagnostic challenges.

## 2. Case Report

This is a consulting case of a 26-year-old Hungarian male patient presenting with an intra-abdominal mass. The computed tomography (CT) showed a relatively circumscribed retroperitoneal lesion measuring 6.3 × 5.7 cm in size. The lesion was closely attached to the pancreatic body, spleen, a segment of colon, and most of the stomach. En bloc resection including distal pancreatectomy, subtotal gastrectomy, splenectomy, and partial colectomy was carried out.

 Macroscopically a well-circumscribed mass measuring 6.5 × 5.5 × 5.0 cm in size was found in the retroperitoneal region with adjacent gastric and colonic wall invasion. It showed a grayish-white cut-surface with focal punctate hemorrhage. The spleen was nearby but free of tumor grossly.

**Figure 1:**
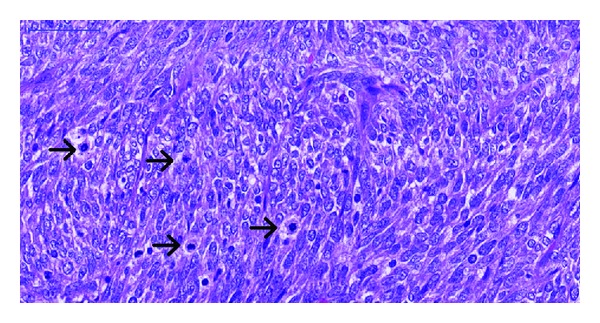
Tumor cells with bland-looing, ovoid to spindle nuclei. Numerous mitotic figures are identified (H&E stain 40x).

**Figure 2:**
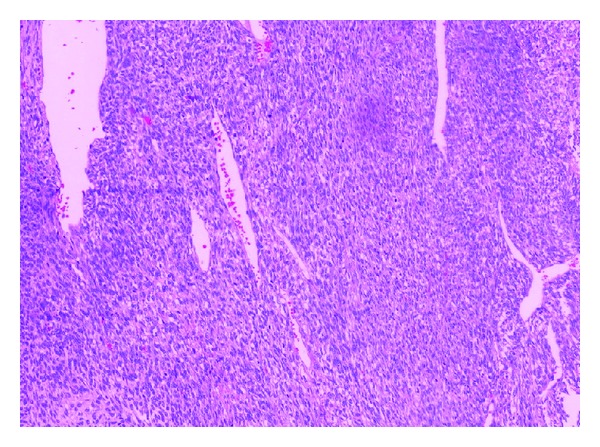
Staghorn vascular structures, similar to the hemangiopericytoma, are seen (H&E 10x).

**Figure 3:**
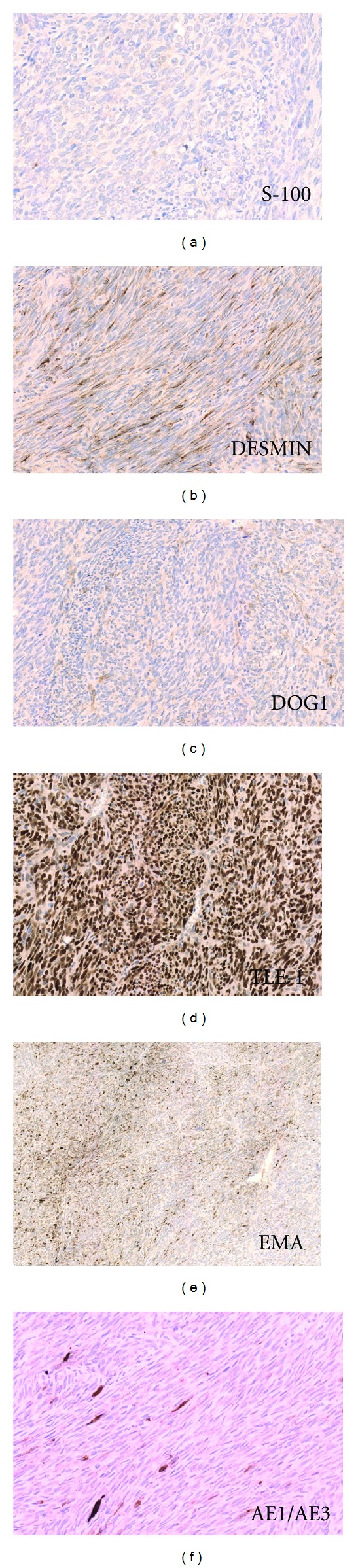
Immunophenotype of tumor cells (tumor cells were focal EMA, AE1/AE3, desmin, and diffuse intranuclear TLE-1 positive; they are negative for S100 and DOG-1).

**Figure 4:**
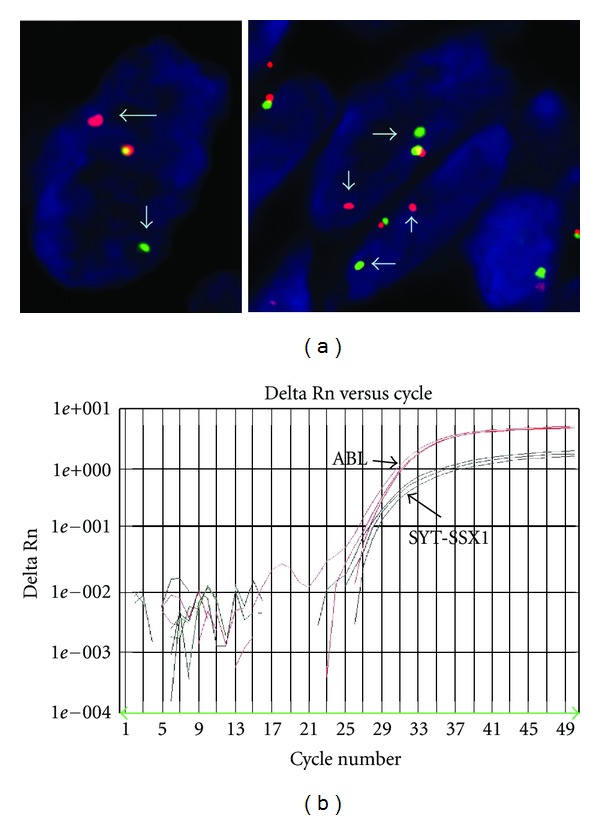
(a) Fluorescent in situ hybridization (FISH) contained mixture of probe-labeled SYT gene. The arrows showed break apart of the gene indicating translocation. (b) Real-time PCR (RT-PCR) revealed the amplification of SYT-SSX1 gene (black); ABL gene (red) was used as internal control.

Histologically the tumor showed spindle to ovoid cells with relatively bland nuclear features forming dense cellular sheets and vague fascicles. Mitoses are numerous (Figure 1). Focal areas show haemangiopericytomatous vascular structures (Figure 2). Tumor cells expressed focal cytokeratin (1 : 150, clone: AE1/AE3, DAKO Cytomation, USA), EMA (1 : 20, clone: sc-9121, Santa Cruz Biochemicals, USA), and, unexpectedly, desmin (clone: DE-R-11, Leica Bond, UK) positivity. Diffuse intranuclear TLE-1 (1 : 20, clone: sc-9121, Santa Cruz Biochemicals, USA) positivity and negativity for CD34 (1 : 300, clone: QBEnd 10, DAKO) and DOG-1 (1 : 200, clone: K9, Novocastra, UK) were also identified (Figure 3). Diagnosis of monophasic synovial sarcoma was confirmed by fluorescent in situ hybridization (FISH) by using a mixture of break-apart probes that contained LSI 5′SYT probe (SpectrumOrange) and LSI 3′SYT probe (SpectrumGreen) (Vysis, USA) to label the 18q11 region in order to demonstrate the translocation of the SYT gene (Figure 4(a)). A real-time polymerase chain reaction was also performed to reveal the SYT-SSX1 fusion gene (Figure 4(b)). We enquired the previous medical history from the original hospital and we found the patient was diagnosed of synovial sarcoma at the right shin 5 years ago and received radiotherapy after the surgery. Pulmonary metastasis was found 3 years after the operation and received wedge resection. We obtained the tissue blocks from the primary and pulmonary metastatic tumor and stained for desmin. The primary tumor showed negative and the pulmonary one revealed focal positive results indicating the possibility of secondary immunophnotype changes of the intra-abdominal metastatic lesion probably due to the previous radiotherapy.

 The postoperative condition of the patient was stable and no further recurrent tumor was found up to the recent followup. 

## 3. Discussion

Synovial sarcoma, a misnomer of possibly mesenchymal cell-derived soft tissue tumor, accounts for 5 to 10% of soft tissue sarcomas with 80% of the cases affecting the extremities around the large joints [3]. Nevertheless, any site may occur. It mainly affects the young adults with a male predominance. Metastatic lesions develop in around half of cases and lung is the most common site. Gastrointestinal synovial sarcoma, either primary or metastatic, is unusual and mainly affects the esophagus [7]. Due to the location, the differential diagnosis is wide. Mainly, gastrointestinal stromal tumor (GIST), myogenic tumor, solitary fibrous tumor, inflammatory myofibroblastic pseudotumor, and neurogenic tumor should be considered. Probably the most important differential diagnosis is GIST, especially CD117-negative cases, since it shares morphological similarities with synovial sarcoma. Membranous DOG-1 positivity in the former and diffuse intranuclear TLE-1 positivity in the latter should tell the difference. Exon sequencing and FISH studies can achieve the definitive diagnosis. Blunt ends and wavy nuclei with S-100 protein positivity are typical for neurogenic tumors; however, differentiating from malignant peripheral nerve sheath tumor (MPNST) can be challenging. Immunostaining for TLE-1 cytokeratin 7/19 and demonstrating the t(X; 18) translocation can solve the difficult cases, since, according to the literature, the MPNST shows only focal weakly TLE-1 positivity instead of diffuse and strong ones seen in synovial sarcoma, and it is usually cytokeratin 7/19 negative [8]. Focal area staghorn vascular structures may simulate a solitary fibrous tumor. CD34 is usually negative in synovial sarcoma. Interpreting the results with caution is important to avoid the diagnostic pitfalls, for example, mast cells within the synovial sarcoma may positively stain for CD117. The characteristic immunophenotype of these tumors is summarized in Table 1.

 Similar ancillary approaches can be applied to differentiate from myogenic tumor; nevertheless, desmin is usually negative in synovial sarcoma [6]. It is known that the recurrent or metastatic tumors may associate with secondary immunophenotype changes. To our best knowledge, metastatic synovial sarcoma associated with desmin positivity, which may mimic myogenic tumor, has not been reported; particularly within the abdominal cavity which may cause diagnostic difficulties. The prognostic factors include the age of the patient, the mitotic activities, and the margin-free resection [9]. Factors still under debate include the histological subtype and the variants of SSX gene involving the translocation. The literature showed that simple and complex karyotypes do correlate with the prognosis. Our previous research data also revealed the correlation between the DNA ploidy, the fine-tuned DNA ploidy, and the high-resolution comparative genomic hybridization (HR-CGH) results. We also found a significant correlation between the different ploidy groups and the clinical outcome [10].

 We reported a case of a metastatic intra-abdominal synovial sarcoma from a 26-year-old man. The histological study showed a monophasic pattern. The tumor cells demonstrated diffuse intranuclear TLE-1 with focal EMA and cytokeratin positivity and DOG-1 negativity. Unusual desmin expression was identified. Fluorescent in situ hybridization and polymerase chain reaction confirmed the diagnosis, with detection of the t(X; 18) translocation and SYT-SSX1 fusion gene, respectively. 

## 4. Conclusion

Intra-abdominal synovial sarcoma, either primary or metastatic, with unusual desmin positivity raised the diagnostic challenge, since a wide range of differential diagnosis could show similar immunophenotype. Typical morphology and focal cytokeratin/EMA positivity should alert to this tumor, and TLE-1, a relatively sensitive marker for synovial sarcoma, should be used to avoid the diagnostic dilemma. FISH and RT-PCR remain the gold standard for the diagnostic confirmation.

## Figures and Tables

**Table 1:** Immunophenotype of the most common mesenchymal tumors in the gastrointestinal tract.

	Synovial sarcoma	GIST	IMT	Leiomyo sarcoma	SFT	Neurogenic tumor
CK/EMA	+	−	−	−	−	−
CD117	−	+	−	−	−	−
TLE-1	+	−	−	−	−	−
h-Caldesmon	−	+/−	−	+	−	−
S100	+/−	+/−	−	−	+/−	+
SMA	+/−	+/−	+	+	−	−
CD34	−	+/−	−	−	+	−
DOG-1	−	+	−	−	−	−
ALK	−	−	+	−	−	−
Desmin	−	−	+	+	+/−	−

GIST: gastrointestinal stromal tumor; IMT: inflammatory myofibroblastic pseudotumor; SFT: solitary fibrous tumor.

## Abbreviation

SS: Synovial sarcoma
GIST: Gastrointestinal stromal tumor
IMT: Inflammatory myofbroblastic pseudotumor
FISH: Florescent in situ hybridization
RT-PCR: Real time-polymerase chain reaction.
